# Effects of porosity on dynamic indentation resistance of silica nanofoam

**DOI:** 10.1038/s41598-017-01152-1

**Published:** 2017-04-21

**Authors:** Cang Zhao, Ying Zhong, Yu Qiao

**Affiliations:** 1 Department of Structural Engineering, University of California – San Diego, La Jolla, CA 92093-0085 USA; 2 X-ray Science Division, Argonne National Laboratory, Argonne, IL 60439 USA; 3 Program of Materials Science and Engineering, University of California – San Diego, La Jolla, CA 92093 USA

## Abstract

The dynamic indentation behaviors of monolithic silica nanofoams of various porosities are investigated. When the pore size is on the nm scale, as the porosity increases, despite the decrease in mass density, the resistance offered by silica nanofoam to dynamic indentation is maintained at a high level, higher than the resistance of solid silica or regular porous silica. This phenomenon is related to the fast collapse of nanocells, which produces a locally hardened region and significantly increases the volume of material involved in impact energy dissipation.

## Introduction

Energy absorption devices (EAD), e.g. armors and protective plates, are usually made of polymers, metals/alloys, or composite materials^1–7^. They provide protection to personnel and important equipment from collision and impact. When the external loading is slow, EAD should be relatively flexible and soft, to conform to the surfaces to be protected; under intense dynamic loadings, EAD must be sufficiently hard^8–10^. For ordinary EAD materials, these two requirements contradict to each other: A soft EAD offers comfort yet its energy absorption capacity tends to be low, and vice versa.

In the past, people studied a variety of concepts of advanced EAD^6, 7, 11–13^. An important EAD material is foam^14–16^. Foam materials, e.g. expanded polymers and porous ceramics and metals, are often processed with foaming agents^17, 18^ or by using templating methods ^19–25^. For protection applications, upon a sufficiently high external loading, the cells collapse. On the one hand, it damages the initial cellular structure; on the other hand, cell buckling leads to a large displacement and dissipates a significant amount of energy. A key parameter of foam is the porosity, *c*. It determines not only the compressibility and the mass density^26^, but also the cell buckling stress, *S*_0_, which on average can be estimated as (1 − *c*)^*δ*^*Y*, with *Y* being the yield strength of ligament material and *δ* being a material constant^27–30^. The energy absorption capacity may be, as a first-order approximation, assessed as *u*_0_ = *S*_0_*c* = *Yc*(1 − *c*)^*δ*^. With a given yield strength (*Y*) and if we assume that *δ* = 1, *u*_0_ is maximized when *c* = 50%. Usually, in engineering practice, the porosity of foams in EAD is in the range from 30% to 70%^31^.

In general, it is believed that the pore size (*d*) of a foam does not have any pronounced influence on its energy absorption capacity, *u*_0_^27–30^. Recently, we carried out a research on nanofoams^32^. When the pore size is on the nanometer (nm) level, the foam behavior under dynamic loading becomes much improved. Firstly, smaller cells collapse faster and thus, the energy absorption process takes effects more rapidly, which is critical to high-strain-rate stress waves. Secondly, and probably more importantly, in a nanofoam, shear localization is suppressed. In a regular foam, as a stress wave propagates, its front can be unstable and the wave energy is concentrated in a few narrow shear bands^33–35^. As a result, the foam fails when the majority part is still undamaged, unable to absorb much energy^32^. Shear banding is associated with local “softening”, as a buckled cell is “weaker” than an unbuckled one. In a nanofoam, because the pore size is relatively small (e.g. smaller than the wave font), cell collapse would cause local compaction, which helps prevent shear bands from further development. Thus, bulk energy absorption is achieved and the protection efficiency is considerably enhanced.

In our previous experiment on silica nanofoams^32^, with a similar porosity and a similar incident stress wave, the transmitted stress wave pressure and energy were much reduced if the nanocell size was smaller than ~200 nm. This finding opens a door to the development of advanced EAD materials. The study was conducted on nanofoam samples with similar porosity ~60%^36, 37^, so as to focus on the pore size effect.

## Results and Discussion

**Figure 1:**
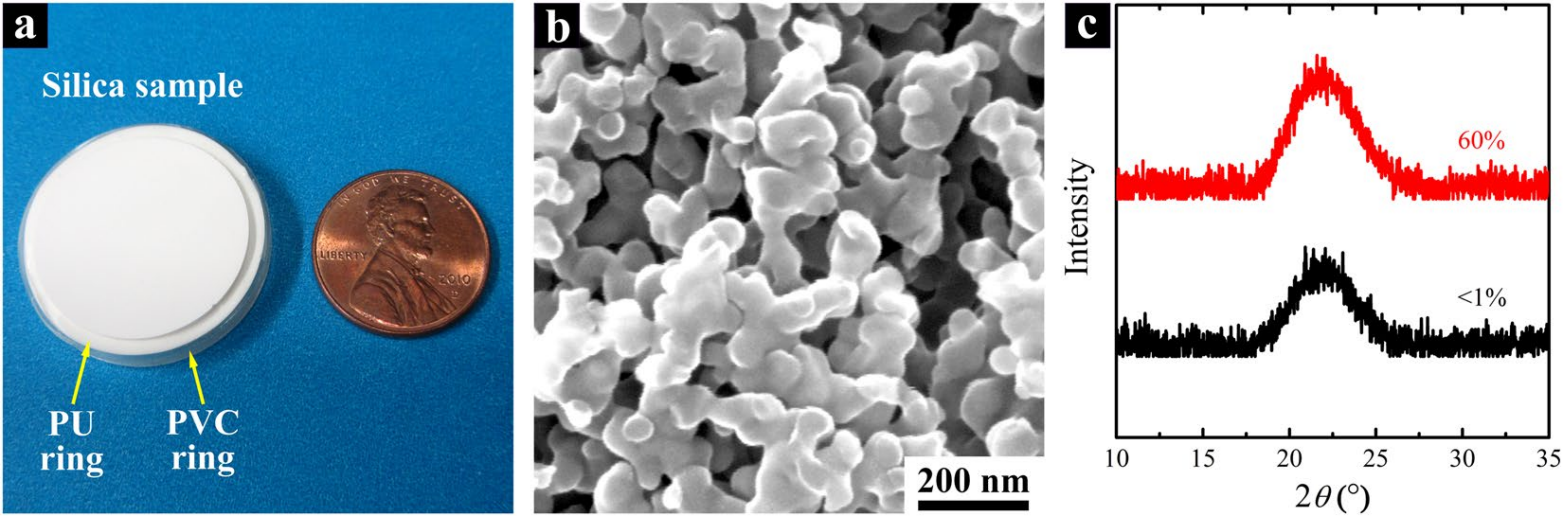
Silica nanofoam. (**a**) A silica nanofoam with a one-cent coin being the reference. The silica disk sample is inserted in a flexible polyurethane (PU) inner ring and a stiff polyvinyl chloride (PVC) outer ring. (**b**) Typical SEM image of silica nanofoam. (**c**) Typical XRD results of silica nanofoam and solid silica; the legend indicates the porosity. The average pore size of silica nanofoam is ~80 nm.

In the current study, we examined the porosity effect on impact indentation resistance of silica nanofoam samples, with the average pore size being kept at ~80 nm. Silica nanofoam monoliths were prepared by a sol-gel approach^17^, and the porous configurations were further adjusted by the subcritical calcination (SCC) technique^36^. When the calcination temperature and duration are in appropriate ranges, compared to the pore size, the porosity is much more sensitive to the temperature. Thus, regardless of the large difference in porosity, the variation in pore size of silica samples with the same initial component mass ratio is much less; for small pores, the variation in pore size can be negligible. In this investigation, the average pore size was measured to be ~80 nm through mercury porosimetry^36^, while the porosity ranged from ~50% to ~70%. Solid silica, employed as the reference material, was fabricated via a similar procedure, whereas the firing was fully conducted to achieve a porosity less than 1%. After polished with sandpapers (silicon carbide, from 320-grit to 2500-grit) to remove the surface layers^38, 39^, the disk-shaped testing samples (Fig. 1a) had the thickness and the diameter of 4.5 mm and ~23 mm, respectively. Figure 1b shows a typical SEM image of silica nanofoam. The cellular channels were random and interconnected. Data of X-ray diffraction (XRD, Fig. 1c) confirmed that all the samples, both nanofoams and solid silica, were in amorphous phase.

**Figure 2:**
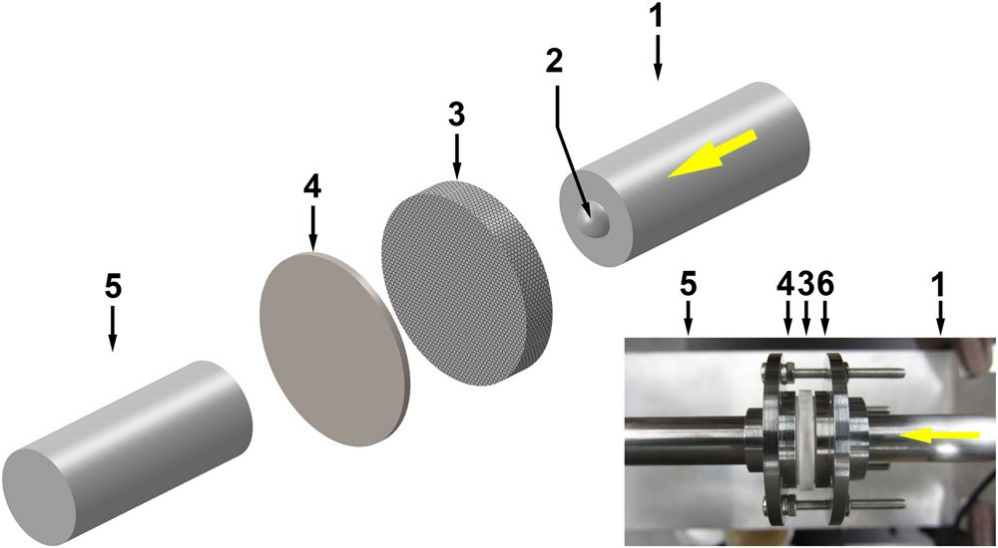
Schematic of dynamic indentation system. The inset shows a photo of mounted silica sample. The numbers indicate: (1) the incident bar; (2) the hemispherical indenter; (3) the silica sample; (4) the steel substrate; (5) the transmission bar; and (6) the front section of the sample holder. In the photo, the indenter cannot be seen.

Dynamic indentation experiments were performed on a custom-made testing system^32, 40^, as illustrated in Fig. 2. A titanium tube was used as the striker, with the total mass being 63 g. It was projected to impact a stainless steel incident bar, by a gas chamber having the initial pressure of 15 psi. The impact speed of the striker was measured to be ~8.5 m/s by a set of EE-SPW421 photo-micro sensors (Omron, Kyoto, Japan). Then, the incident bar impacted a tungsten carbide (TC) indenter (Bal-tec, Los Angeles, CA) into the silica sample. The indenter was hemispherical-shaped, with the diameter of 4.75 mm. Its hardness was more than 91 HRA and its maximum surface roughness was 0.7 micro-inch Ra. For the silica sample, two rings were used to confine its lateral surface (Fig. 1(a)): The inner ring was made of relatively flexible polyurethane (PU), with the initial inner radius of 9.5 mm and the outer radius of 11 mm; the outer ring was made of polyvinyl chloride (PVC), with the initial inner radius of 12.5 mm and the shrinkage ratio of 2:1. The silica sample was placed on a substrate and a transmission bar; both of them were made of the same stainless steel as the incident bar. The impact striker, the incident bar, and the transmission bar had the same 12.7 mm diameter. In the middle of the incident and the transmission bars, a pair of strain gauges (WK-13-250BF-10C, Vishay Measurements Group) were attached, and a data acquisition system (2310B, Vishay Measurements Group) was employed to record the stress wave signals. In order to preserve the sample for further examination, only the first stress wave pulse was allowed to impact the silica sample, which was realized by a momentum trapper^32, 41, 42^. As shown by the inset in Fig. 2, the symmetrical configuration of the rear and the front parts of the dynamic indentation system could effectively reduce sample bending^32^.

**Figure 3:**
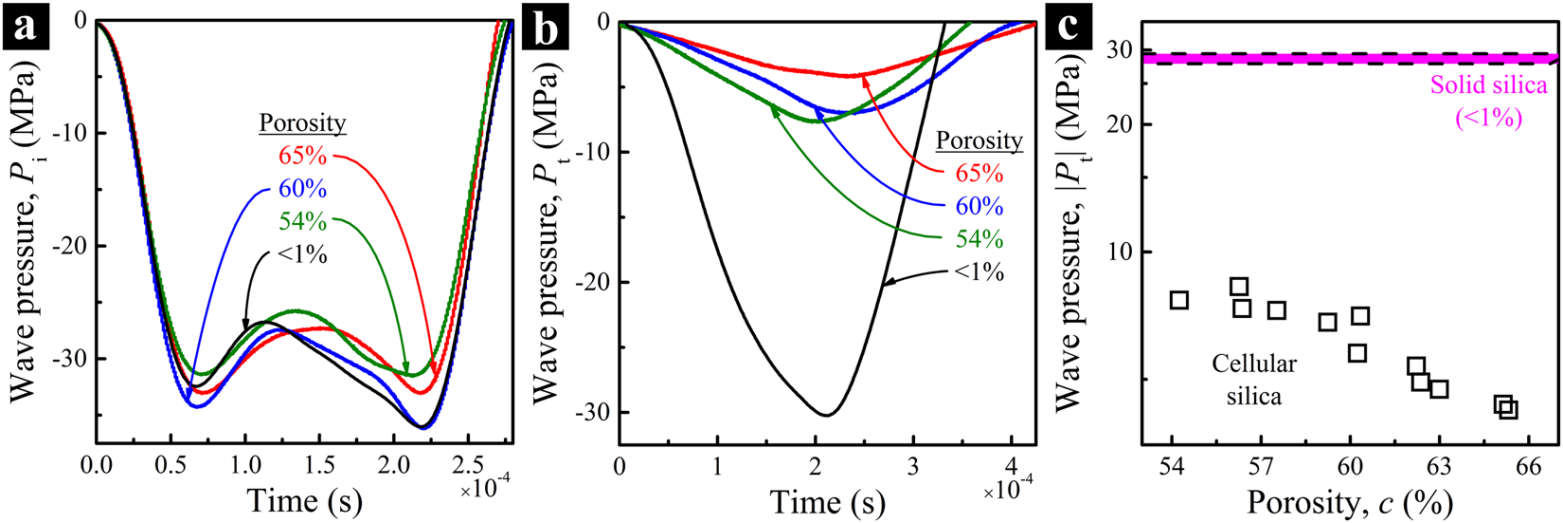
Typical stress wave signals: (**a**) Incident waves and (**b**) transmitted waves. (**c**) Peak pressure of transmitted wave as a function of porosity; the purple band with dashed boundaries indicates the range of peak pressures of transmitted waves of solid silica (*c* < 1%). For all the silica nanofoam samples, the average pore size is ~80 nm and the striker speed is ~8.5 m/s.

Figure 3 shows typical stress wave signals of the silica samples. Upon similar incident stress waves (Fig. 3(a)), for the samples with different porosities, the transmitted stress waves (Fig. 3(b)) vary significantly, owning to the difference in acoustic impedance and possibly also energy absorption^32, 43^. The relationship between the peak transmitted wave pressure, |*P*_t_|, and the porosity, *c*, is displayed in Fig. 3(c). Compared with solid silica wherein *c* < 1%, |*P*_t_| of silica nanofoam is much lower; with the increase in porosity from ~50% to ~70%, |*P*_t_| decreases from ~8 MPa to ~4 MPa.

**Figure 4:**
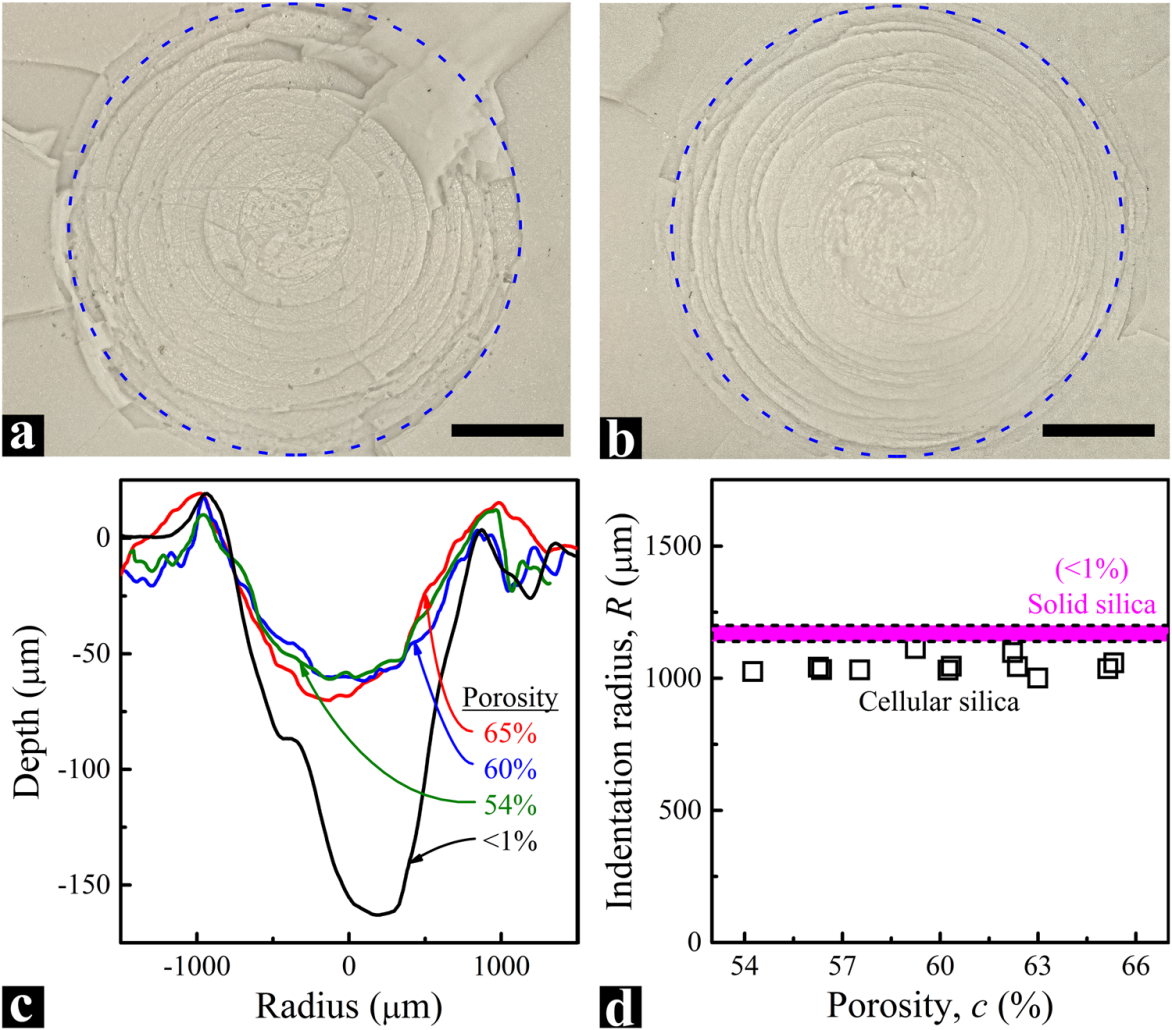
Indented surfaces of silica nanofoams with the porosities of (**a**) 54% and (**b**) 65%, respectively. The blue dashed circles indicate the indentation boundaries. The scale bars are 500 μm. (**c**) Typical indentation depth profiles. (**d**) Indentation radius, *R*, as a function of porosity, *c*. The purple band with dashed boundaries shows the indentation radius range of solid silica (*c* < 1%). For all the silica nanofoam samples, the average pore size is ~80 nm and the striker speed is ~8.5 m/s.

After the impact tests, the samples were examined by a VHX-1000 microscope. Through the 3D Image Stitching function, the indentation profiles were scanned, as displayed in Fig. 4. On the sample surface (Fig. 4(a) and (b)), the indentation radius was measured (Fig. 4(d)). Under the dynamic loading, with the porosity increasing from ~50% to ~70%, the maximum indentation depth (*D*) and radius (*R*) are maintained around 60 µm and 1050 μm, respectively; both are smaller than those of solid silica. The data suggest that silica nanofoam is effectively “harder” than solid silica, and the effective dynamic hardness is relatively unrelated to the porosity; that is, the empty nanocells do not weaken the dynamic indentation resistance, in the range of porosity under investigation. Compared to the maximum indentation depth, the difference in indentation radius between solid silica and silica nanofoam is smaller, implying that after indentation, along the depth direction the cellular structure has partially recovered.

Based on the classical theory^28–30^, the hardness, *H*, of a foam material should be highly dependent on its porosity, *c*:

1 $$H\propto {(1-c)}^{\delta }$$

With the increase in porosity or decrease in mass density, the foam material should become softer and exhibit less resistance to indentation. In general, the hardness of a material can be evaluated as^44, 45^

2 $$H=\zeta \frac{F}{\pi {R}^{2}}$$

where *F* is the indentation force, *R* is the indentation radius, and *ζ* is a geometric factor. Therefore, for a foam material, under a given loading,

3 $$1/{\bar{R}}^{2}\propto {(1-c)}^{\delta },$$

where $\bar{R}=R/{R}_{{\rm{solid}}}$ is the normalized indentation radius, and *R*_solid_ is the indentation size of solid silica under the same loading. Clearly, for a regular foam, the indentation radius should increase with the porosity. However, as shown in Fig. 4(d), this classical $\bar{R}$-*c* relationship breaks down for silica nanofoams. The unique phenomenon may be employed to reduce the weight of an EAD without losing indentation resistance under dynamic loadings.

For self-comparison, we define an index to describe the nominal indentation resistance (NIR):

4 $$\Theta =1/{\bar{R}}^{2}$$

**Figure 5:**
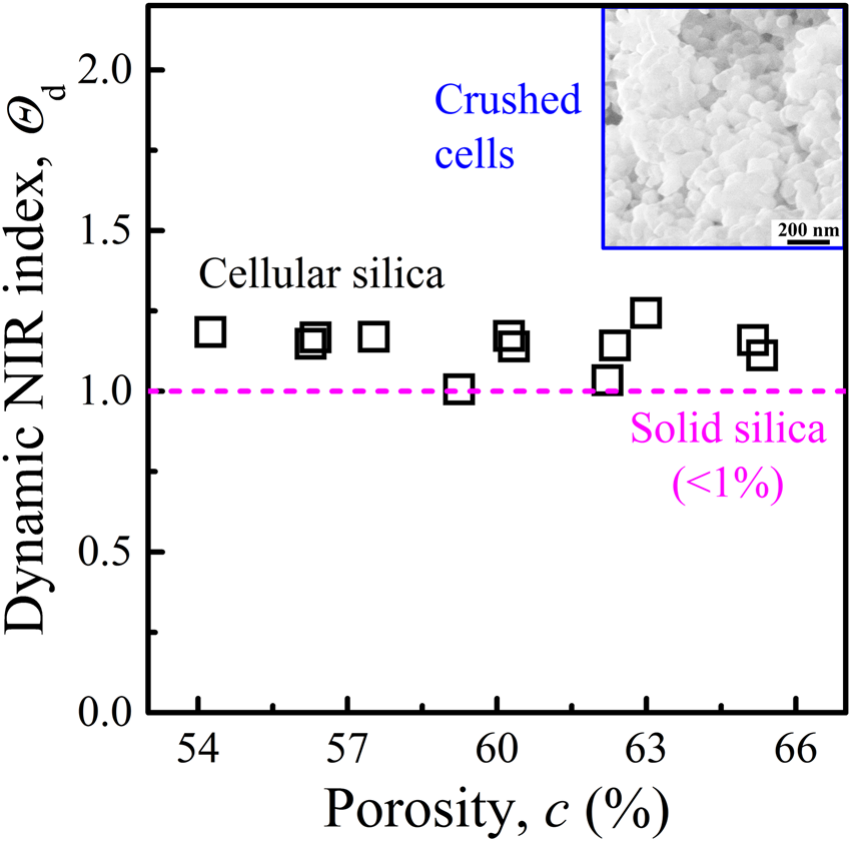
The index of nominal indentation resistance (NIR) under dynamic loading. The dashed horizontal line indicates *Θ*_d_ of solid silica (*c* < 1%). The inset shows typical crushed cells. For all the silica nanofoam samples, the average pore size is ~80 nm and the striker speed is ~8.5 m/s.

NIR index under dynamic loading will be denoted as *Θ*_d_ in the following discussion. The reduction in normalized indentation size leads to the increase in the NIR index. In Fig. 5, with the increase in porosity, *Θ*_d_ fluctuates around 1.13. It should be related to the fast collapse of nanocells^32, 46^, as illustrated by the inset in Fig. 5. The crushing of nanocellular structure results in a locally hardened region at the front of indenter, which renders a much larger volume of material being involved in resisting the indenter motion.

## Conclusions

To conclude, for silica nanofoam with the average pore size around 80 nm, under dynamic loading, when its porosity increases from ~50% to ~70% and its mass density reduces by ~40%, the indentation resistance is maintained at a similar level, higher than that of its solid counterpart and much higher than that of regular porous silica. The maximum transmitted wave pressure decreases rapidly as the porosity increases. The underlying physical mechanism is credited to the fast collapse of nanocells at the front of indenter, which forms a locally hardened layer and therefore, a large volume of material contributes to indentation resistance.

## Methods

### Materials

Silica nanofoam samples were obtained through sol-gel processing^17^, and the subcritical calcination (SCC) technique was adopted to adjust their porous configurations^37^. First, with the mass ratio of 72:28 and the total weight of 800 g, Kasil-1 potassium silicate (PQ Corporation, Valley Forge, PA) and Ludox HS-40 silica sol (Sigma-Aldrich) were mixed for 30 min. Then, under vigorously stirring, 200 g formamide solution (40 wt%) was added. After stirring for another 30 min, it was poured into polypropylene (PP) plastic vials. The diameter and the height of the vials were 36 mm and 16 mm, respectively. At room temperature (RM), the mixture was aged for 24 hours. After phase separation, the gels were washed by a series of solutions at 90 °C: 1M ammonium nitrate, 1M nitric acid, and deionized water; they were then rinsed by methanol at RM for more than five times. Next, the gels were dried in a vacuum chamber for 72 hours at 80 °C. Finally, the samples were calcinated in a tube furnace (GSL-1700X, MTI Corporation, USA) for one hour at different temperatures slightly above the glass transition temperature of amorphous silica, ~1200 °C. As the reference material, solid silica samples were prepared through a similar procedure, except that in the last calcination step they were fired at 1250 °C for 12 hours, to achieve a porosity less than 1%.

### Pore size

The pore size of nanofoam was assessed through mercury porosimetry^47^: *d* = 4*σ*·cos*θ*/*P*_Hg_, where *σ* is the surface tension of mercury (0.484 N/m at RM), *θ* is the contact angle (140°), and *P*_Hg_ is the infiltration pressure of mercury.

### Porosity

The porosity of nanofoam sample was estimated from the measured mass density (*ρ*): *c* = 1 − *ρ*/*ρ*_solid_, where *ρ*_solid_ is the mass density of amorphous silica (2.2 g/cm^3^).

### SEM analysis

Before and after the tests, scanning electron microscope (SEM) analysis was performed with a FEI-XL30 SEM at 20 kV. Prior to the examination, the samples had been coated with iridium under argon atmosphere for 6 seconds at 85 mA using a K575X sputter coater (Emitech, Houston, TX).

### X-ray diffraction

The foam and solid silica samples were inspected on a Bruker D8 Advance X-ray Diffractometer with Cu Kα radiation (wavelength 1.5418 Å). The 2*θ* range was set to be 10–80°; the speed was 0.1 sec step;^−1^ the step size was 0.02°.

### Impact system

Both the incident and the transmission bars were made of 17-4 PH H900 stainless steel^32^, which has the mass density of 7.75 g·cm^−3^ and the elastic modulus of 196.5 GPa. The diameters of the two bars were 12.7 mm, and the lengths were 1.8 m and 1.5 m, respectively. The impact striker was a Grade 9 Titanium (Ti) tube, having the inner diameter, the outer diameter, and the length of 11.4 mm, 12.7 mm, and 462 mm, respectively. Its two ends were sealed using two 17-4 PH H900 stainless steel end-caps (thickness = 5 mm), respectively, under the assistance of two stainless steel pins with the diameter and the length of 3.2 mm and 12.7 mm, respectively. The total weight of the striker, end-caps, and pins is 63 g.

### Indentation profile scanning

A Keyence VHX-1000 digital microscope (Osaka, Japan) was used to characterize the indented samples. Through the function of 3D Image Stitching, the indentation profiles were scanned, with the scanning range spanning across the lowest and the highest focusing points. The scan step size was set to be less than 2 μm.

## References

[1] Hull D (1991). A unified approach to progressive crushing of fibre-reinforced composite tubes. Composites science and technology.

[2] Simone AE, Gibson LJ (1998). Aluminum foams produced by liquid-state processes. Acta Materialia.

[3] Saafi M, Toutanji H, Li Z (1999). Behavior of concrete columns confined with fiber reinforced polymer tubes. ACI materials journal.

[4] Gibson LJ (2000). Mechanical Behavior of Metallic Foams. Annual Review of Materials Science.

[5] Gaitanaros S, Kyriakides S (2015). On the effect of relative density on the crushing and energy absorption of open-cell foams under impact. International Journal of Impact Engineering.

[6] Tancogne-Dejean T, Spierings AB, Mohr D (2016). Additively-manufactured metallic micro-lattice materials for high specific energy absorption under static and dynamic loading. Acta Materialia.

[7] Hawreliak JA (2016). Dynamic behavior of engineered lattice materials. Scientific Reports.

[8] Abramowicz W, Jones N (1986). Dynamic progressive buckling of circular and square tubes. International Journal of Impact Engineering.

[9] Frommeyer G, Brux U, Neumann P (2003). Supra-ductile and high-strength manganese-TRIP/TWIP steels for high energy absorption purposes. ISIJ international.

[10] Ebrahimi H, Ghosh R, Mahdi E, Nayeb-Hashemi H, Vaziri A (2016). Honeycomb sandwich panels subjected to combined shock and projectile impact. International Journal of Impact Engineering.

[11] Liu L, Chen X, Lu W, Han A, Qiao Y (2009). Infiltration of electrolytes in molecular-sized nanopores. Physical Review Letters.

[12] Xu B, Chen X, Lu W, Zhao C, Qiao Y (2014). Non-dissipative energy capture of confined liquid in nanopores. Applied Physics Letters.

[13] Yang W (2016). Large-deformation and high-strength amorphous porous carbon nanospheres. Scientific Reports.

[14] Nyce GW, Hayes JR, Hamza AV, Satcher JH (2007). Synthesis and characterization of hierarchical porous gold materials. Chemistry of materials.

[15] Tappan BC, Steiner SA, Luther EP (2010). Nanoporous metal foams. Angewandte Chemie International Edition.

[16] Vukovic I, ten Brinke G, Loos K (2013). Block copolymer template-directed synthesis of well-ordered metallic nanostructures. Polymer.

[17] Shoup, R. D. In *Colloid and Interface Science* Vol. 3 (ed. Milton Kerker) 63–69 (Academic Press, 1976).

[18] Nakanishi K (1997). Pore structure control of silica gels based on phase separation. Journal of Porous Materials.

[19] Kuang D, Brezesinski T, Smarsly B (2004). Hierarchical porous silica materials with a trimodal pore system using surfactant templates. Journal of the American Chemical Society.

[20] Sha J, Gao C, Lee S-K, Li Y, Zhao N (2016). Preparation of three-dimensional graphene foams using powder metallurgy templates. ACS nano.

[21] Ashley, C. E. *et al*. The targeted delivery of multicomponent cargos to cancer cells by nanoporous particle-supported lipid bilayers. *Nat Mater* **10**, 389–397, http://www.nature.com/nmat/journal/v10/n5/abs/nmat2992.html#supplementary-information (2011). 10.1038/nmat2992 PMC3287066 21499315

[22] Wu KC-W, Yamauchi Y (2012). Controlling physical features of mesoporous silica nanoparticles (MSNs) for emerging applications. Journal of Materials Chemistry.

[23] Lee YC, Chen CT, Chiu YT, Wu KCW (2013). An effective Cellulose‐to‐Glucose‐to‐Fructose conversion sequence by using Enzyme immobilized Fe3O4‐loaded mesoporous silica nanoparticles as recyclable biocatalysts. ChemCatChem.

[24] Titze T (2015). Microimaging of transient concentration profiles of reactant and product molecules during catalytic conversion in nanoporous materials. Angewandte Chemie International Edition.

[25] Yamamoto E, Kuroda K (2016). Colloidal mesoporous silica nanoparticles. Bulletin of the Chemical Society of Japan.

[26] MacMinn CW, Dufresne ER, Wettlaufer JS (2016). Large deformations of a soft porous material. Physical Review Applied.

[27] Maiti SK, Gibson LJ, Ashby MF (1984). Deformation and energy absorption diagrams for cellular solids. Acta Metallurgica.

[28] Gibson, L. J. & Ashby, M. F. *Cellular solids: structure and properties*. (Cambridge university press, 1997).

[29] Scheffler, M. & Colombo, P. *Cellular ceramics: structure, manufacturing, properties and applications*. (John Wiley & Sons, 2006).

[30] Hodge AM (2007). Scaling equation for yield strength of nanoporous open-cell foams. Acta Materialia.

[31] Dunand DC (2004). Processing of titanium foams. Advanced engineering materials.

[32] Zhao C, Qiao Y (2016). Fast-condensing nanofoams: Suppressing localization of intense stress waves. Materials Science and Engineering: A.

[33] Balch DK (2005). Plasticity and damage in aluminum syntactic foams deformed under dynamic and quasi-static conditions. Materials Science and Engineering: A.

[34] Dannemann KA, Lankford J (2000). High strain rate compression of closed-cell aluminium foams. Materials Science and Engineering: A.

[35] Tan P, Reid S, Harrigan J, Zou Z, Li S (2005). Dynamic compressive strength properties of aluminium foams. Part I—experimental data and observations. Journal of the Mechanics and Physics of Solids.

[36] Zhao C, Wang M, Shi Y, Cao J, Qiao Y (2016). High-temperature post-processing treatment of silica nanofoams of controlled pore sizes and porosities. Materials & Design.

[37] Zhao C, Qiao Y (2016). Characterization of nanoporous structures: from three dimensions to two dimensions. Nanoscale.

[38] Diao Ying, Harada Takuya, Myerson Allan S., Alan Hatton T., Trout Bernhardt L. (2011). The role of nanopore shape in surface-induced crystallization. Nature Materials.

[39] Kawaguchi T, Iura J, Taneda N, Hishikura H, Kokubu Y (1986). Structural changes of monolithic silica gel during the gel-to-glass transition. Journal of Non-Crystalline Solids.

[40] Kolsky, H. *Stress waves in solids*. Vol. 1098 (Courier Corporation, 1963).

[41] Nemat-Nasser S, Isaacs JB, Starrett JE (1991). Hopkinson techniques for dynamic recovery experiments. *Proceedings of the Royal Society of London*. Series A: Mathematical and Physical Sciences.

[42] Song B, Chen W (2004). Loading and unloading split Hopkinson pressure bar pulse-shaping techniques for dynamic hysteretic loops. Experimental Mechanics.

[43] Surani FB, Kong X, Panchal DB, Qiao Y (2005). Energy absorption of a nanoporous system subjected to dynamic loadings. Applied Physics Letters.

[44] Luo J, Stevens R (1999). Porosity-dependence of elastic moduli and hardness of 3Y-TZP ceramics. Ceramics International.

[45] Oliver WC, Pharr GM (2004). Measurement of hardness and elastic modulus by instrumented indentation: Advances in understanding and refinements to methodology. Journal of materials research.

[46] Leonard A, Daraio C (2012). Stress wave anisotropy in centered square highly nonlinear granular systems. Physical review letters.

[47] Washburn EW (1921). The dynamics of capillary flow. Physical Review.

