# Publisher Correction: Effects of porosity on dynamic indentation resistance of silica nanofoam

**DOI:** 10.1038/s41598-018-35444-x

**Published:** 2018-11-21

**Authors:** Cang Zhao, Ying Zhong, Yu Qiao

**Affiliations:** 1 Department of Structural Engineering, University of California – San Diego, La Jolla, CA 92093-0085 USA; 2 X-ray Science Division, Argonne National Laboratory, Argonne, IL 60439 USA; 3 Program of Materials Science and Engineering, University of California – San Diego, La Jolla, CA 92093 USA

Correction to: *Scientific Reports* 10.1038/s41598-017-01152-1, published online 21 April 2017

The original version of this Article contained errors in the spelling of the authors Cang Zhao, Ying Zhong and Yu Qiao which were incorrectly given as Zhao Cang, Zhong Ying & Qiao Yu.

These errors have now been corrected in the PDF and HTML versions of the Article.

